# Editorial: Rocks, Plants and Microbes

**DOI:** 10.3389/fpls.2021.745338

**Published:** 2021-08-25

**Authors:** Camille Eichelberger Granada, Bruno Britto Lisboa, Luciano Kayser Vargas

**Affiliations:** ^1^Programa de Pós-graduação em Biotecnologia, Universidade Do Vale Do Taquari – Univates, Lajeado, Brazil; ^2^Laboratório de Química Agrícola, Departamento de Diagnóstico e Pesquisa Agropecuária (DDPA), Secretaria Estadual da Agricultura, Pecuária e Desenvolvimento Rural, Porto Alegre, Brazil

**Keywords:** biological weathering, crushed rocks, plant growth-promotion, soil amendment, carbon sequestration

Tropical and subtropical soils are, in a general sense, highly weathered acidic, dystrophic, and mineralogically poor. Weathering, many times associated with naturally poor parent material, has left these soils depleted not only of nutrients, but also of chemically active secondary mineral. The situation is often exacerbated by nutrient leaching and soil erosion caused by unsustainable agricultural practices.

In this context, stonemeal techniques, based on the application of crushed rocks and their dissolution by the action of plant roots and microbial communities, can be an alternative to traditional methods for soil amelioration. Rock powders can be a source of plant nutrients, can raise soil pH and can also alter soil mineralogy, resulting in geological rejuvenation and improved soil fertility, due to the increase of cation exchange capacity. Moreover, the beneficial effects of stonemeal are not only restricted to the local sphere, as it affects the carbon balance. This Research Topic discusses how plants and microbes interact to decompose rock minerals, affecting soil fertility and mineralogy, crop productivity and carbon balance.

The excessive use of chemical fertilizers raises the production costs and generates environmental concerns, such as lixiviation and depletion of P and K sources (Basak et al., 2017; Granada et al., 2018). In this context, the review article authored by Ribeiro et al. brings the importance of rhizospheric processes and biological weathering (siderophores and organic acids production by microorganisms) for enhancing mineral dissolution and, consequently, providing nutrients for plants in a sustainable way. The authors highlighted the importance of selection and characterization of new bacterial strains capable of decomposing crushed rocks, aiming to increase soil fertility and improve crop production when they are used as inoculants. The highly sustainable use of silicate rocks, solubilized by microbial inoculants or by the natural soil microbiota, can also contribute to the reduction of climate changes and decrease the dependency of developing countries on imported chemical fertilizers (Adegbeye et al., 2020). Some subjects covered by this review were confirmed in the findings of Ding et al. These authors cultivated sweet potato plants in rocky soil, in China, without and with low N fertilization. The low N fertilization improved total and available N, available P and K, and microbial nitrogenase and urease activities. Bacterial diversity was reduced in root environment subjected to low nitrogen fertilization. However, the relative abundance of P and K-solubilizing bacteria, N-fixing and urease-producing bacteria increased, while the relative abundance of plant pathogens decreased. At the end of the crop cycle, sweet potato yield increased by half. Thus, low-dose N fertilization favored the development of plant growth-promoting bacteria present in the soil, increasing biological weathering and nutrient availability for plants. This cleaner strategy seems to be a good option for environmentally friendly agriculture (Chandrakala et al., 2019).

Different types of alkaline-earth minerals can be used as soil amendment, depending on their regional availability. Casas et al. studied microbial induced calcite precipitation in sandy soil, using dolerite as Ca source. The data showed large CO_2_ emissions when dolerite fines were applied, almost twice of that observed in the treatment without mineral application. However, inorganic carbon precipitation was also higher in dolerite treatment. At the end, the authors conclude that dolerite fines had a large but short-lived carbon sequestration potential. Haque et al. added wollastonite in the crop of two leguminous plants, soybean and alfalfa inoculated with *Bradyrhizobium japonicum* and *Sinorhizobium meliloti*, respectively, and evaluated atmospheric CO_2_ sequestration and storage as inorganic C in agricultural soils. The addition of wollastonite increased the soybean yield and alfalfa growth. The rate of CO_2_ sequestration achieved 0.08 kg CO_2_·m^−2^·month^−1^ (~3.0 g of CaCO_3_ Kg^−1^) in alfalfa, and the formation of some weathering products emphasized the importance of the rhizosphere environment to degrade rocks and available nutrients. These strategies of soil amendment would encourage farmers to use silicate minerals in soil, helping to contribute toward global climate change mitigation, without compromising crop yield.

Carbon dioxide sequestration, its mineralization into carbonates and elemental cycling by oxalate-carbonate pathway were discussed in the mini-review presented by Syed et al. Oxalotrophic bacterial communities are ubiquitous in soils, and they can oxidize the oxalate, forming carbonates. The authors draw attention to the fact that soil instability, caused by loose compaction of soil particles and the loss of water holding capacity, are the foremost reason for desertification phenomena. To fight this process, the CO_2_ precipitation by oxalotrophic bacterial populations provides the binding of sandy grains, increasing soil stability and creating a soil carbonate reserve, restoring the soil balance and fertility. Physiological and biochemical processes can also be increased by long-term soil application of lime and phosphogypsum in no tillage systems, as demonstrated by Bossolani et al. The long-term soil effects were the reduction of soil acidity, the increase of the availability of P, Ca^2+^, Mg^2+^, and ${\text{SO}}_{4}^{2-}$–S, and the improvement of maize plant nutrition, increasing the concentration of photosynthetic pigments and reducing oxidative stress, which led to higher grain yield.

Thus, this Research Topic brings new insights into the use of rock powders to improve soil fertility, plant productivity, and environmental quality, and shows how microbes play a central role in these processes. We hope this the collection of articles will stimulate new discussions and encourage new research on this subject of increasing interest and importance.

## Author Contributions

All authors drafted the content of this document, revised, and approved the final version of the editorial text for submission.

## Conflict of Interest

The authors declare that the research was conducted in the absence of any commercial or financial relationships that could be construed as a potential conflict of interest.

## Publisher's Note

All claims expressed in this article are solely those of the authors and do not necessarily represent those of their affiliated organizations, or those of the publisher, the editors and the reviewers. Any product that may be evaluated in this article, or claim that may be made by its manufacturer, is not guaranteed or endorsed by the publisher.
